# The perils of pay variability: Determinants of worker aversion to variable compensation in low- and middle-wage jobs

**DOI:** 10.1371/journal.pone.0347194

**Published:** 2026-05-26

**Authors:** Lindsey D. Cameron, Jirs Meuris

**Affiliations:** 1 Wharton School, University of Pennsylvania, Philadelphia, Pennsylvania, ‌‌United States of America; 2 School of Business, University of Wisconsin, Madison, Wisconsin, ‌‌United States of America; University of Crete, GREECE

## Abstract

A substantial proportion of the labor force in low- and middle-wage jobs is prone to pay variability, or variance in earnings from paycheck to paycheck. Emerging research suggests that pay variability can be detrimental to workers’ well-being and enhance their likelihood of exiting their job. Using several iterations of qualitative and quantitative data, we identify and evidence critical determinants of workers’ experience of pay variability and their likelihood of voluntary turnover. Combining insights from exploratory interviews and surveys with ride-hailing drivers (N = 63) and cognitive appraisal theory, we predicted that pay variability is most likely to result in voluntary turnover when (a) workers were more frequently earning lower-than-average paychecks, (b) felt limited control over their earnings, and (c) their household was dependent upon their paycheck. Using matched survey and archival data from a sample of truck drivers (N = 711) from a national transportation company, we subsequently found that pay variability is positively associated with the likelihood of turnover, but this relationship significantly varied with the predicted contextual moderators. Additional quantitative analyses demonstrated that this relationship was observed even among the highest performing drivers and, surprisingly, likely resulted in less annual pay for those who left. Supplemental qualitative data provided further support for the predicted moderators. Taken together, our findings explain why and when variable compensation represents a source of precarity for low-and middle-wage workers that can motivate turnover. Theoretical and practical implications are discussed.

## Introduction

In recent decades, employers across industries and sectors have replaced the principles of reciprocity and long-term commitment that characterized employment relationships with a short-term, transactional approach focused upon maintaining flexibility and containing labor costs [1–3]. As part of these changes, many employers abandoned their role as the bearer of risk in the employment relationship [4,5], shifting the burden of market fluctuations away from shareholders onto the shoulders of individual workers [6]. One of the primary ways that employers have minimized risk to shareholder returns is through variable compensation systems that enable adjustments in labor costs alongside the ebbs and flows of the market [6,7]. Variable compensation systems transfer market risk onto workers by linking performance incentives to criteria that fluctuate based on market forces [8,9] or by adopting work arrangements that explicitly modify paychecks according to market fluctuations, such as variable scheduling (e.g., [10–12]). A national survey by [13] found that over 60% of large employers use compensation in which a proportion of workers’ paychecks is tied to fluctuating inputs. Ostensibly, this implies that two workers may have similar annual earnings yet experience divergence in the volatility of its acquisition.

Past research has focused predominantly on how organizations might benefit from variable compensation systems by allowing firms to adjust for labor costs and insulate profits from market shocks (e.g., [14–16]). However, understanding the outcomes of variable compensation systems also requires us to consider individual workers’ experiences [17]. Indeed, [18] noted that work practices are often evaluated entirely from the vantage point of the employer with limited regard for workers’ perspective where a “dark side” may emerge. Despite variable compensation offering workers the opportunity to earn more when they meet specific performance criteria and/or when market conditions are positive and demand is high, these benefits may often be outweighed for low- and middle-wage workers, defined as U.S. workers who earn up to twice the median wage or approximately $70,000 or less per year [19]. These workers are more likely to occupy jobs that result in pay variability [20]—defined here as variance in earnings from paycheck to paycheck *within the same job*—and the proportion of this population experiencing pay variability is growing [21].

Studies suggest that pay variability can often disrupt low- and middle-wage workers’ lives [22–26] and be detrimental to their health [27] because most lack a safety net [28] and rely on small frequent paychecks to meet their needs [29,30]. Conroy and colleagues [31] show that the negative consequences of pay variability on these workers will extend to organizations by increasing the likelihood of voluntary turnover. However, pay variability can represent a profoundly different experience depending on the context of the worker and the job, and thus by extension the degree to which people are motivated to quit is likely to diverge. This means that a comprehensive understanding of the “dark side” associated with variable pay systems would benefit from a framework to explain variance in low- and middle-wage workers’ experience of pay variability and who is more likely to choose to leave their job in response.

Using multi-sourced qualitative and quantitative data from two occupations, we extend the emerging literature on the experience and outcome of pay variability to identify critical moderating conditions. To gain a deeper understanding of workers’ lived experiences, we first conducted exploratory semi-structured interviews and collected survey data from ride-hailing drivers, a set of low- and middle-wage workers who are prone to high levels of pay variability. We observed that pay variability can be a highly stressful experience, one that many strive to mitigate by continuously adjusting their behavior at work or finding other employment. The exploratory data pointed towards cognitive appraisal theory (CAT; [32,33])—which outlines the factors that shape individual reactions to stressful experiences—as a useful foundation for developing predictions on when individual workers would be more averse to pay variability and more motivated to leave their job. Drawing from our qualitative data and CAT, we predicted that the relationship between pay variability and voluntary turnover would be determined by three conditions: (a) the relative frequency of lower-than-average paychecks, (b) whether a worker believes that they have the ability to control their earnings, and (c) the availability of alternative sources of income within a worker’s household.

Our predictions were supported using matched archival and survey data from a sample of full-time truck drivers employed by a U.S. transportation company. Supplemental quantitative analyses suggested that those who left with high pay variability included high-performing, highly skilled drivers, indicating that pay variability was motivating valued workers to leave. These analyses further showed that workers with high levels of pay variability were unlikely to be leaving for a higher paying job or a sign-on bonus, as they were earning significantly more than the median truck driver in their state. Supplemental qualitative analyses provided further support for our theoretical arguments highlighting threat perceptions as the mechanism underlying variance in the relationship between pay variability and voluntary turnover.

Altogether, our paper contributes to the broad literature focused on the implications of risk-shifting from employers onto workers. While prior research has provided insight into the organizational mechanisms underlying the Great Risk Shift [4,6], it has only begun to unpack the consequences for individuals. Emerging research suggests that fluctuations in compensation can harm well-being among low- and middle-wage workers (e.g., [22,25,27]) and increase their likelihood of voluntary turnover [31], but what factors determine workers’ response to these fluctuations remain an open question. Drawing on qualitative and quantitative data across two samples, this paper introduces a novel framework rooted in cognitive appraisal theory to explain *why* and *when* pay variability among low-to-middle wage workers represents an aversive experience that can result in turnover. Within this framework, we identify three contextual moderators—the shape of the paycheck distribution, feelings of control, and paycheck dependence—that determine when pay variability will increase the likelihood of voluntary turnover. In doing so, our research indicates that there are long-term costs for employers associated with the transfer of market risk onto workers through variable compensation systems but also identifies potential avenues for employers to limit turnover costs. We conclude with practical implications about designing variable compensation systems that benefit employers and workers.

## Variable compensation in contemporary organizations

Since the mid-1970s, traditional employment relationships, defined by long-term commitment between employers and their workers, have been steadily replaced with transactional short-term employment relationships [1,34]. Traditional employment relationships were marked by relative stability in worker compensation because a steady living wage was construed as a long-term investment that ensured optimal workforce productivity [35]. Employers, rather than workers, generally absorbed the risks associated with fluctuating market conditions such as product availability and consumer demand to provide a fixed paycheck [3]. However, with the rise of shareholder activism in the late 1970s, employers looked to abdicate their role as the primary risk bearer in the employment relationship [4,5]. As a result, fixed compensation became construed as a threat to profits and shareholder returns, which encouraged the adoption of variable compensation systems that align labor costs with market conditions [6].

Employers can align labor costs with market forces using variable compensation systems in two ways. Market-influenced performance incentive metrics are based on fluctuations in external trends [8], such as consumer demand and product availability [3,13]. Batt and Colvin ([9]), for example, describe how performance incentives for call center workers can be based on the number of calls serviced, which depends upon the number of customers that call during one’s shift. Likewise, performance pay among salespeople can be constrained by consumer demand, as well as by product availability and pricing [36]. Second, pay can fluctuate alongside market factors when the work arrangement is explicitly adjusted for them. Variable scheduling, for instance, allows employers to alter work hours from week to week depending upon consumer demand, and workers’ compensation will fluctuate depending on how many work hours they are allocated in a given week (e.g., [10,11]). Similarly, workers’ pay may vary based on the volume of work available. Two prominent examples of these arrangements are commercial truck drivers who are paid based upon the characteristics of the load they transport (e.g., size of load, material transported) and flight attendants who are paid based upon the characteristics of the flight they work (e.g., flight length, cabin of service). Workers within the gig economy, such as Uber or Instacart, also fall in this category as they are only “hired” when tasks are available and compensated on a per-task basis [37–39]. In sum, employers can adjust compensation for fluctuation in market forces either indirectly through the selection of criteria for performance incentives or directly through the design of their work arrangements.

With the increasing prevalence of variable compensation systems, a substantial number of low- and middle-wage workers now occupy jobs where they are prone to pay variability [40–42]. An analysis of 250,000 bank accounts by the J.P. Morgan Institute found that approximately one-third of households vary in their income from month to month with 86% of the volatility in income was driven by fluctuations in earnings within the same job [43]. Similarly, surveys collected by the Federal Reserve Board [28] identify income volatility among one-third of respondents, with approximately 60 percent of those working full-time for an employer. Pay variability accounts for a significant proportion of volatility in household income. In a multi-year financial diary study of 235 low-to-middle income households, Morduch and Sewicki [42] found that within-job variability in earnings underlies the largest proportion of volatility in household income and spells of episodic poverty. Providing insight into which groups are most likely to experience pay volatility from financial diaries, Hannagan and Morduch [20] found the highest levels of volatility among low- and middle-wage households earning up to 300 percent of the poverty line (approx. $79,500 for a family of four). Morris et al. [21] show that income volatility significantly increased for low-to-middle income households over the last 25 years while declining for upper-income households.

## A qualitative exploration of low- and middle-wage workers’ experience of pay variability

To gain insight into low- and middle-wage workers’ experience of pay variability, we first collected interview and survey data from a sample of ride-hailing drivers in North America. Ride-hailing drivers are an ideal or “extreme case” [44] for theory development around the experiences surrounding pay variability, because as independent contractors in a closed labor market, they are especially prone to experiencing pay variability. Pay is algorithmically determined and fluctuates based on various inputs such as the timing of their shift, demand, and tips, as well as incentives [44]. Interviews were conducted by the first author consisting of longitudinal semi-structured interviews (N = 107) with 63 drivers in 23 North American cities. Interview and survey data was collected between August 31, 2016 and Feb 20, 2020. All interviewees provided verbal consent and those who also completed surveys provided written consent. Per the stipulation of our IRB agreement, this data is not available publicly. Drivers were asked general questions about their work and specific questions about their finances. All interviews except one were conducted in English and all interviews except eleven were professionally transcribed. One interview was conducted in French and transcribed by first author. Another participant declined to be audio-recorded and in the other cases the audio files became corrupted. Participants’ data was recorded and analyzed based on the contact summary sheet [45] that was created immediately after each interview. At the time of drivers’ first interview, tenure ranged from two weeks (10 rides) to seven years (18,000 rides) with an average of 14 months and 1,800 trips.

Each driver was asked to complete a survey to collect demographic details as well as descriptive data regarding their work, pay, and household composition. Thirty-eight drivers (86% of second-round interviewees) completed the survey. The majority of respondents reported that they were their household’s sole financial provider (62.5%) with at least one person financially dependent on them (M = 0.96, SD = 1.26). Over a 6-week period, respondents, on average, earned $497.78 (SD = 407.46) and drove 9.8 (SD = 22.41) hours per week. The upper cap of a middle-wage earner’s annual income is $70,000 [19] which corresponds to $1,350 a week. Thus, we considered ride-hailing drivers as low- to middle-income earners. However, there was considerable variance in their earnings from week to week, as indicated by the within-person standard deviation across the six weeks reported in the survey (M = 115.91, SD = 126.01). Importantly, while fluctuations in work hours per week (M = 39.83, SD = 22.42) were significantly correlated with variability in earnings (*r* = .449, *p* < .05), work hours only explained 20.1% of their pay variability, indicating that it was not exclusively driven by variation in the amount of time spent working. We analyzed the qualitative data using grounded theory techniques [46]) and coded around the themes of pay and the contextual factors that seemed to influence it*.* In the following sections, we highlight the insights that emerged from the interviews.

### The Burden of pay uncertainty

Our first insight was that workers were struggling to ensure that their paychecks were sufficient to meet their financial needs. Most drivers noted that they were attracted to the ride-hailing industry because of the potential for higher earnings, but many quickly realized “that not every day is going to be a great day money wise” (20) in part because they “are not guaranteed [consistent high pay] when it is slow and [or] not in the right area” (28). Earnings in ride-hailing can vary considerably because they are based upon fluctuating mileage rates [47]. Indeed, drivers complained that the work was “not as lucrative as it once was” (7) because rates changed daily (“On some days it’s really good, especially when it’s surging, but on days when it’s not surging it’s a waste of time and money” (46)) and seasonally (“I was hitting them [incentives] in the winter … whereas now that it’s getting nicer out… it’s a lot harder because everybody wants to walk on nice days instead of taking an Uber” (44)). Noting the pay fluctuations, one driver said, “This is the only job where you can go to work and do the exact same thing every day and it costs you money [you make less money]” (4). These daily and weekly fluctuations in earnings left drivers “stressed” (20) and “pissed” (3) because they often had to scramble and unexpectedly drive longer than anticipated when they did not earn as much as they normally did in a given shift.

Over time, some drivers decided that the only way to effectively relieve the strains associated with income uncertainty was to leave the industry altogether. Some left for secure municipal jobs, becoming a bus driver (20), a medic (59), or an admin (64). One worker, who left to become a delivery driver for a fast-food chain, noted that he had “definitely overworked himself” while attempting to ensure he earned enough every week (3). In sum, our interviews suggested that workers often view pay variability as an aversive experience because it can lead to uncertainty in whether their earnings will be sufficient to meet their income needs. Therefore, some decided to leave for alternative employment opportunities that could offer more income stability.

### Perceived control over paychecks

We further observed differences in the extent to which drivers felt they could effectively control their earnings through their own efforts. While some participants adopted elaborate strategies to influence their earnings, others felt less able to do so, viewing the app and algorithm as the sole determinants of their paychecks. Describing the randomness of the algorithm in assigning and pricing rides, one driver noted that, “Driving is like a box of chocolates, you never know where the good ride is at” (10). Alternatively, respondents who felt more confident in their capacity to control their earnings across paychecks labelled themselves as “planners” (48), “systematic” (28), and “drilling down” (20) in how they structured their time and waiting locations.

Drivers sought to exert control over their earnings to meet their household’s financial needs using a combination of strategies including goal setting, pre-positioning in high-demand areas, and chasing incentives. Oftentimes, drivers set their own daily and/or weekly goals, which represented the minimum amount to meet their household’s needs. As one driver explained, “I’d like to say [I stop driving] when I’m somewhat fatigued, but if I haven’t reached that goal, I fight off fatigue and get rejuvenated. The closer I get to the goal; I get extra energy. My goal is $75 a day” (45). Strategizing about how to meet their financial needs and even out their paychecks, drivers would wait at certain locations to optimize the chances of being matched by the algorithmic management system to high-yield rides: “I hang around hotels in downtown Detroit. There is an area where you can sit between three hotels. …To me, it’s just about putting yourself in the right position at the right time” (4). Another strategy drivers deployed was to chase the algorithmically determined incentives that appeared during high-demand times, such as completing a consecutive number of rides within a few hours or a specific number of rides over a few days. Some drivers synchronized when, where, and for how long they would drive with these incentives in the hopes of a steadier paycheck, noting, for example, that they “try to be in the area where the incentives are going to be at as that’s more money” (11). The time and energy these strategies required alongside the uncertainty of how long it would take to “make a buck” (7) took a toll. When one part-time driver, who was his family’s breadwinner, was asked if he considered switching to driving full-time, as he often earned more per-hour driving, he replied negatively, highlighting the uncertainty in earnings: “I wouldn’t like it at all because then you’re worried about making enough money to make it” (21). And some drivers simply couldn’t drive any more. Noting they were physically “already at [their] max” (64), one driver kept their expenses low and lived where they worked—their car. It is important to note that driving longer hours was not always a means to eradicate pay variability. Starting in 2018, drivers were only allowed to work twelve hours on a platform and then had to wait eight hours before logging in again. Moreover, some drivers were unable to work more due to physical constraints or other responsibilities.

### Alternative sources of income

Another factor that influenced the experience of pay variability was whether workers had a stable alternative source of household income. Drivers from households with multiple sources of income were less likely to consider the uncertainty about their earnings as adverse. One driver, whose executive spouse’s income comprised the majority of their household income, noted, “My wife is the breadwinner and I just drive while she’s at work, and other than that I’m a stay-at-home-dad. I take care of the kids” (55). Similarly, another driver mentioned that when his income from driving was less than expected, his family of three relied upon his wife’s steady income as a teacher which was “a blessing” (22). In these cases, other income sources served as a financial safety net when paychecks were lower than expected, ensuring that the household’s ability to meet its financial needs were never threatened.

Conversely, pay variability appeared to be a more aversive experience among workers whose household income was highly dependent upon their paychecks. When paychecks were less than expected or there was a spike in expenses, participants reported having to ‘drive hard’ or turn to other jobs. In part based of the sampling technique of the larger qualitative study ([37,38]) and Pareto’s 80/20 rule, for most of the interviewed drivers ride-hailing was the primary source of income for most drivers. (See [48] for additional context about the high levels of economic dependence among workers in the gig economy.) A recent immigrant and breadwinner for a family of seven complained about his workday increasing from eight to twelve hours after mileage rates dropped to cover his bills; he eventually moved his family two hours away, where he thought there would be less competition from other drivers (20). A single mother was frustrated that after rate cuts and decreased demand she now had to drive many more hours than she used to—sometimes up to fourteen hours a day—every time her teenage daughter wanted money for a special event or ran up the water bill (9). Extra hours added mental and physical stress to those who were their families sole income providers. While some drivers simply drove longer to smooth over the pay variability, other drivers supplemented their income with other sources. In the summer, when demand in his college town dropped, one breadwinner augmented with agricultural work (21) while another turned to hairdressing (46). The fact that drivers could simply drive longer to make up for any income shortfalls was a benefit of being an in a piece-rate system in which work was (nearly) always available. Even so, workers who depended on ride-hailing earnings as a stable source of income viewed pay variability as a more aversive experience.

## A Contextual model of pay variability and voluntary turnover Among low- and middle-wage workers

**Fig 1:**
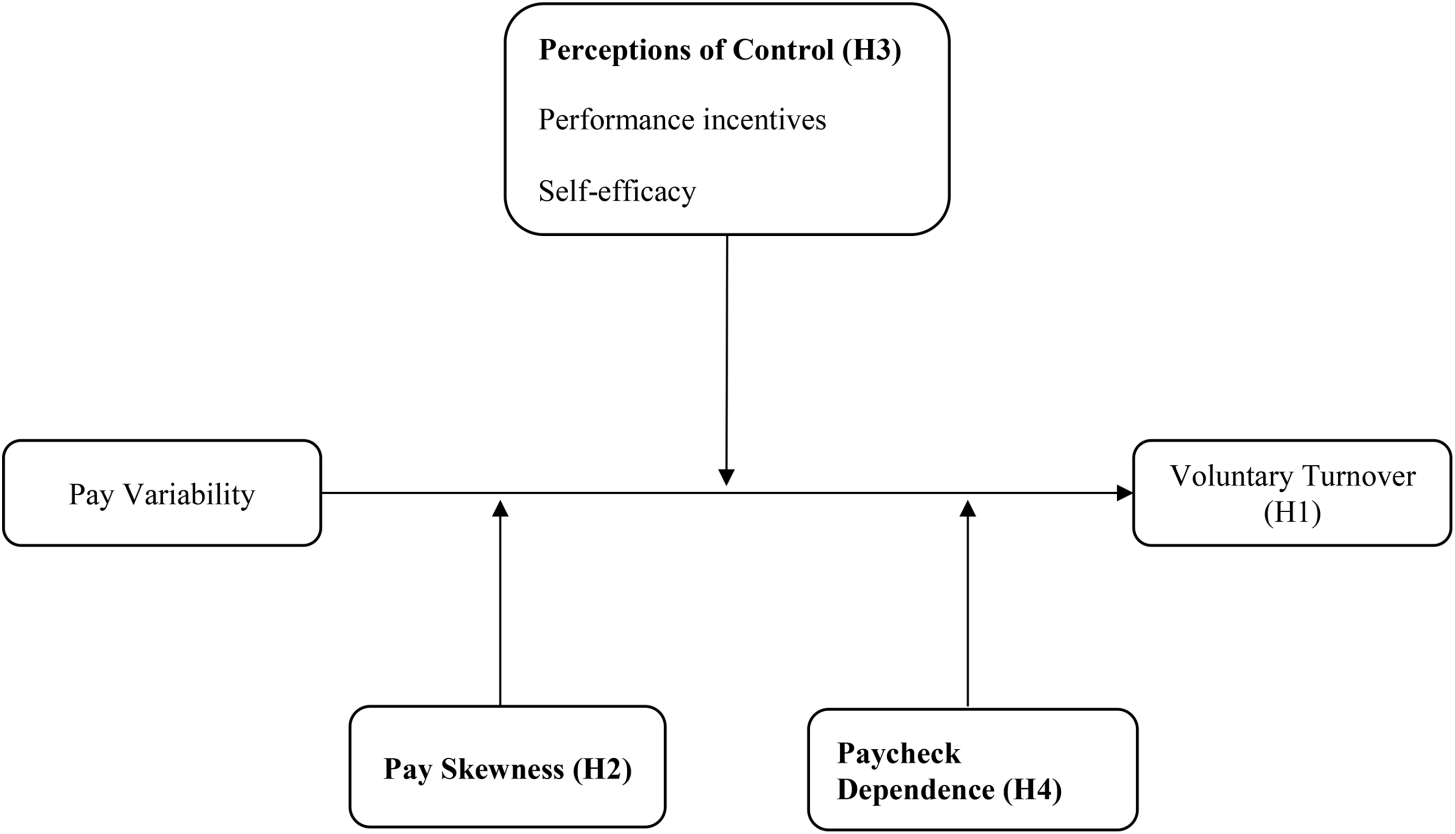
A Model of the Relationship Between Pay Variability and Voluntary Turnover Among Low- and‌‌ Middle-Wage Workers.

The exploratory data indicated several factors that will shape the degree to which pay variability represents an aversive experience for low- and middle-wage workers. Among a sample of workers who experience high levels of pay variability, ride-hailing drivers, we found that workers were repeatedly striving to mitigate their pay variability, and that a sizable portion ultimately sought to distance themselves from pay variability by leaving the job, even if they would be earning less. Relying upon our exploratory findings, we subsequently developed a conceptual model (see Fig 1) rooted in cognitive appraisal theory (hereafter referred to as CAT; [32,33]) to explain *why* and *when* pay variability is most likely to push low- and middle-wage workers towards the exit. That is, in our exploratory study, we suggested individual differences in how workers viewed their fluctuating compensation, and CAT provides a framework to explain why individuals may differ in their appraisal of the same experience. A long-standing theory in psychology, CAT argues that variance in the response to a stressful experience like pay variability originates from how people appraise it “in terms of its personal relevance and in terms of their abilities or options for coping” [49]. Given an event or situation, they engage in a primary appraisal process (is this situation threatening or harmful to me?) and a secondary appraisal process (do I have the resources and coping capacity to manage the demands?) [50]. These appraisals represent subjective evaluations that may deviate from objective reality but determine emotional responses and how people respond to the situation. Stronger negative reactions occur when individuals appraise situations as a threat to their well-being and they do not perceive to have the capacity to effectively cope with it [51]. As such, we identify primary and secondary appraisals as central to low- and middle-wage workers’ experiences of pay variability and predicting when it motivates them to leave their job.

### Voluntary turnover in response to pay variability

In our interviews with ride-hail drivers, we observed aversion to pay variability when it threatened their ability to meet the financial needs of their household. As a result, drivers were constantly devising strategies to mitigate the threat, such as prepositioning in certain areas and chasing surges. Despite deploying these strategies, a proportion of drivers ultimately left for other jobs with more stable paychecks. Higher levels of pay variability often present a threat to low- and middle-wage workers and their households because it becomes more difficult to predict whether there will be enough financial resources available to satisfy needs. Although economic models suggest that people will update their savings and consumption patterns to adjust for variation across paychecks [52], low- and middle-wage workers usually have limited capacity to adjust for economic shocks. Downward fluctuations in earnings for low- and middle-wage workers can lead to financial strain [22,25,42]. Indeed, studies have linked pay variability to utility disruption [23], food insecurity [53], missed bill payments [54], and mortgage delinquency [24]. As a result, the uncertainty of extensive pay variability is more likely to be viewed by low- and middle-wage workers as a threat to their household’s well-being that they are unable to effectively cope with, increasing their motivation to create distance from the uncertainty by leaving their job. Fulmer and Shaw [55] posit this will enhance the salience of self-protective motives and increase the likelihood of sorting out in response to pay variability as they “seek to preserve, increase, and stabilize future income” (p. 943). Taken together, and building on prior research [31], the baseline prediction of our model is a positive relationship between pay variability and voluntary turnover.

**Hypothesis 1.** Pay variability is positively associated with the likelihood of voluntary turnover.

### Contextual moderators

***Shape of Paycheck Distribution*** Our exploratory data suggested that the adverse experience of pay variability among low- and middle-wage workers is driven by the prospect of their earnings falling short of financial needs. Since low- and middle-wage workers tend to have limited capacity to adjust for shortfalls [56], they must rely on their higher-than-average paychecks to provide the resources to absorb any shortfall that might occur with lower-than-average paychecks. However, a high level of variability across paychecks does not inherently mean that low- and middle-wage workers experience a balanced frequency of higher-than-average and lower-than-average paychecks. Instead, lower-than-average and higher-than-average paychecks can vary in their relative frequency, as indicated by the shape of their pay distribution. Even when two workers earn the same on average, the pattern of how their pay fluctuates around that average can be very different. With more frequent lower-than-average paychecks, a worker’s distribution clusters more often below the mean with occasional large spikes above it. On the other hand, with more frequent higher-than-average paychecks, paychecks cluster above the mean with few, but sharper, dips.

Whether paychecks more frequently fall above vs. below average earnings should have divergent consequences for the impact of pay variability on workers. Some evidence for this is provided by Christelis et al. [57] who found that transient losses in income are more consequential for consumption behavior than equivalent gains in income. For workers with high levels of pay variability who more frequently receive paychecks below their average earnings, they are more likely to have negative secondary appraisals feeling that they do not have the capacity to effectively cope with the uncertainty associated with pay variability [58]. Under these circumstances, the baseline experience is one of shortfall rather than adequacy. Since frequent lower-than-average paychecks means workers will have more pay periods where their earnings are below expectations, it will require them to more often confront their available financial resources being less than their financial needs. Regularly receiving less than expected forces workers into a constant state of financial vigilance, heightening concerns about meeting recurring expenses such as rent, food, and utilities. In this pattern, the rare high-pay weeks feel like exceptions that cannot be relied upon, doing little to offset the ongoing strain created by frequent deficits. This leads to feelings of helplessness in effectively dealing with pay variability. In contrast, among low- and middle-wage workers who more commonly earn paychecks that exceed their average, the uncertainty of pay variability should be seen as less of a threat. When pay variability is anchored in mostly higher-than-average paychecks with only infrequent dips, workers experience a more stable sense of sufficiency where the occasional low-pay week is likely to be interpreted as a manageable setback that they can effectively cope with rather than a chronic, insurmountable threat.

Taken together, the shape of a person’s paycheck distribution will alter the secondary appraisal of pay variability. Given the same level of pay variability, workers with more frequent lower-than-average paychecks are more apt to feel helplessness in effectively dealing with it whereas workers with more frequent higher-than-average paychecks will see it as more manageable. According to cognitive appraisal theory (CAT; [50]), differences in secondary appraisals will explain variance between workers in experienced strain and likelihood of turnover under the same level of pay variability. This leads to our second prediction that the relationship between pay variability and voluntary turnover should depend on the relative frequency of higher-than-average paychecks to lower-than-average paychecks.

**Hypothesis 2***.* The shape of the pay distribution moderates the positive relationship between pay variability and the likelihood of voluntary turnover, with a stronger relationship among workers whose paychecks more frequently fall below their average.

***Perceived control.*** Another source of variance that emerged from our exploratory data was the extent to which workers believed that they could control their paychecks. This is consistent with the secondary appraisal process proposed by Cognitive Appraisal Theory where people are less likely to feel strain from a potentially threatening situation when they believe that they can mitigate it [51], but also extensive research in organizational psychology showing that strenuous facets of a job are experienced as less aversive when people believe they have the latitude and capacity to effectively respond and deal with it [59]. Therefore, we anticipate that the positive relationship between pay variability and voluntary turnover will be weaker among workers who believe they can individually manipulate their earnings. We propose two factors that should independently influence workers’ feelings of control over their paychecks: (a) the source of pay variability (performance incentives vs. non-performance factors) and (b) perceptions of self-efficacy.

While many low- and middle-wage workers derive their earnings predominantly from variable inputs, there are significant differences in how variable compensation systems are structured. Pay variability originates from variance in performance metrics, non-performance inputs such as variable schedules, or a combination of both. Integrating expectancy theory [60] helps explain why the *source* of pay variability can determine workers’ secondary appraisal of their ability to cope with it. Expectancy theory posits that motivation at work is a function of three cognitive components: expectancy (the belief that effort leads to performance), instrumentality (the belief that performance leads to valued rewards), and valence (the value individuals place on those rewards). Central to the theory is that workers must believe that outcomes are contingent on their own actions rather than external or arbitrary factors. When a larger proportion of pay variability stems from performance-based incentives, workers experience a heightened sense of control [61], as their outcomes are seen as more tightly connected to their outcomes, reinforcing both expectancy and instrumentality beliefs. Because performance incentives endow low- and middle-wage workers with a sense of perceived control over their paychecks, they should have more positive secondary appraisals of the uncertainty associated with pay variability since they “are able to manipulate the rewards they receive by varying their performance levels” [62]. Conversely, when fluctuations in pay result from non-performance-related factors such as variable task assignments or scheduling changes, workers are more likely to have negative secondary appraisals of pay variability. These workers feel that there is less they can do to avert downward fluctuations and ensure their earnings are sufficient for their financial needs. Under these conditions, the negative secondary appraisals will increase the strain experienced with pay variability, heightening the motivation to leave in response. Hence, we predict that the relationship between pay variability and voluntary turnover will be attenuated among those workers for whom a greater proportion of their paychecks originates from performance incentives.

**Hypothesis 3a.** Performance incentives moderate the positive relationship between pay variability and the likelihood of voluntary turnover, with an attenuated relationship among workers who derive a greater proportion of a paycheck from performance incentives.

Beyond performance incentives, perceived control over earnings may also originate from individual differences in self-efficacy, defined as a person’s “belief about their capabilities to exercise control over their own level of functioning and over events that affect their lives” [63]. Individuals with higher levels of self-efficacy tend to believe that they can overcome obstacles in their environment through their own actions [64,65]. Bandura [66] argued that low self-efficacy “create stress by diverting attention from how best to proceed with the undertaking to concerns over failings and mishaps” (p. 123) while individuals with higher self-efficacy “deploy their attention and effort to the demand of the situation and are spurred to greater effort by obstacles” (p. 123). Consistently, individual differences in self-efficacy will also shape whether workers feel they have the capacity to enact control over their work. Importantly, these beliefs represent subjective evaluations of one’s personal capabilities that are distinct from objective conditions of control. Even when jobs offer people limited autonomy, stronger self-efficacy beliefs lead workers to perceive themselves as capable of working effectively within the confines of their latitude and/or attaining more autonomy through their own efforts, leading them to be less frustrated or distressed by it.

Workers with high levels of self-efficacy will tend to have positive secondary appraisals and believe that they can control their earnings sufficiently to meet their financial needs using on-the-job strategies, which will buffer the strain of uncertainty in earnings [59]. Uncertainty appears more tolerable to people when they see themselves as able to deal with the demands [67]. As a result of these positive secondary appraisals, pay variability should be less likely to increase the likelihood of those with a high level of self-efficacy from leaving their organization. On the other hand, those who lack self-efficacy will have negative secondary appraisals of pay variability because they see themselves as less able to control it. They are more likely to frame the uncertainty of pay variability as an unmanageable threat to their well-being [67], increasing the experienced strain of this demand [59]. Indeed, there is some evidence that self-efficacy tends to decrease people’s dislike of working under variable compensation systems [68]. Therefore, we predict that self-efficacy will attenuate the relationship between pay variability and voluntary turnover.

**Hypothesis 3b.** Self-efficacy moderates the positive relationship between pay variability and the likelihood of voluntary turnover, with an attenuated relationship among workers who have high self-efficacy.

***Paycheck dependence.*** Our exploratory data further pointed towards a third contextual moderator influencing low- and middle-wage workers’ aversion to pay variability: paycheck dependence, defined as the proportion of the focal worker’s household income that is dependent upon their paycheck. Though organizational research focused on workers’ financial context has been limited, it can profoundly shape their work behavior because most people rely on their employment earnings to fulfill their household’s financial needs [69]. Paycheck dependence can affect how people view their paychecks because any change in earnings directly impacts their household [70–72]. However, workers will differ in the degree to which their households rely on their paychecks. Whereas some households are largely reliant on one breadwinner’s paycheck, other households are able to combine multiple streams of income [73].

Consistent with CAT, we anticipate that paycheck dependence affects secondary appraisals of pay variability where workers whose household is highly dependent on their paychecks are more likely to see paycheck dependence as a demand they are unable to cope with. A high level of pay variability among workers presents more opportunities for earnings to fall short of financial needs because there are few alternative sources of income for the household to rely on. Under these conditions, frequent drops in earnings have a greater potential to endanger the household’s welfare, which should result in a stronger motivation among highly dependent workers to address the variability in their income. Some jobs such as ride-hailing in our exploratory study allow workers to work longer hours to smooth out any pay variability while those in other jobs can only increase their income by taking on a second job. However, our exploratory findings indicated that this is often unsustainable over time, and thus workers with high paycheck dependence will eventually become motivated to distance themselves from pay variability. In contrast, workers whose households are less dependent on their paycheck will be less likely to perceive pay variability as an unmanageable threat because there are alternative streams of income available in the household that serve as a safety net for when earnings within a paycheck or a few consecutive paychecks fall short of financial needs. Hence, our final prediction is a stronger relationship between pay variability and voluntary turnover among workers whose household is highly dependent upon their paycheck.

**Hypothesis 4.** Paycheck dependence moderates the positive relationship between pay variability and the likelihood of voluntary turnover, with a stronger relationship among workers whose household is highly dependent on their paycheck.

## Methods

### Trucking Co

We examined our hypotheses using matched survey and archival data from a sample of commercial truck drivers employed by Trucking Co., a large transportation company operating across the United States. At Trucking Co., drivers receive a weekly paycheck based upon their work from the prior week. Several factors influenced drivers’ paycheck. Akin to the majority of companies in commercial trucking [74], load assignments at Trucking Co. were algorithmically determined and compensation was task-based [75]. That is, pay was calculated based upon the characteristics of the load (e.g., weight, hazardous material), the distance driven, and the number of stops. Performance incentives (e.g., no at-fault accidents, surpassing the miles per gallon (MPG) benchmark), area-specific supplements (e.g., delivering in urban areas), and pay for non-driving time (e.g., (un)loading,) could also boost weekly earnings. Consequently, drivers’ earnings could fluctuate across paychecks depending upon variance in all these factors. Even within the same business unit (e.g., dedicated lines), there was considerable divergence in pay variability as the assigned loads fluctuated from week-to-week. Thus, our context offers an opportunity to examine voluntary turnover among a group of low- to middle-wage workers in the same organization engaging in a similar task with varying levels of pay variability.

### Data collection

Data used for the analyses were derived from a survey and HR records. Data was collected from May 1, 2018 to December 30, 2019 and all participants provided written consent. Data was fully anonymized before the research team accessed it. Per the stipulation of our agreement with Trucking Co., this data is not available publicly. We first administered an online survey to full-time truck drivers employed by the company, excluding drivers who were in training or worked as independent operators (contractors). We focused on full-time drivers to ensure that all workers in our sample were comparable in terms of load (task) assignment and pay schemes, as both vary considerably for independent contractors. Of the 4,300 drivers eligible to participate, 711 completed the survey, a 16.5% response rate. Our response rate is comparable to other survey research with this population of workers (e.g., 7.8% - [76]; 19.3% - [77]). While our response rate is on the lower end of typical survey response rates in management studies [78], long-haul truck drivers are an atypical population. Long-haul truck drivers are dispersed and on-the-road while working, constraining the ability to complete web-based surveys due to lack of time, space, and/or internet connection. Furthermore, even though they worked as employees for a company, the lack of embeddedness with their employer also limits their motivation to respond to survey requests even if it is supported by their employer.

Examination of available population characteristics revealed that our sample offered a good representation of the overall driver population: turnover (26.4% in the sample vs. 19% in the population), average performance pay ($205 per month in the sample vs. $182 per month in the population) and non-performance pay ($4,204 per month in the sample vs. $3,984 per month in the population), miles per gallon benchmark attainment (80.1% of weeks in the sample vs. 74.7% of weeks in the population), and accident rate (16% with at least one accident in the sample vs. 13% with at least one accident in the population). For each driver who completed the survey, we received their HR records directly from the company for the six months before and after survey administration. These records included demographic information, weekly paycheck amounts, weekly workload, and performance. We matched this data to the survey responses using confidential identifiers. Table 1 reports the summary statistics and correlations amongst the variables included in the analyses.

**Table 1:** Summary Statistics and Correlations.

	Variables	M (SD) or %	1	2	3	4	5	6	7		9	10	11	12	13	14	15
1	Pay average	1,049.34 (268.70)															
2	Pay variability	282.20 (111.19)	.472														
3	Pay skewness	‒.048 (1.05)	‒.132	.025													
4	Performance incentives	3.26 (2.29)	.177	.104	‒.129												
5	Self‒efficacy	5.72 (.98)	‒.001	.112	.036	‒.012											
6	Paycheck dependence	79.73 (22.84)	.003	‒.015	.051	.012	‒.032										
7	Household partner	65.80%	.134	.053	.001	.053	.012	‒.413									
8	Multiple jobs	2.7%	.005	‒.012	‒.022	‒.066	‒.029	‒.093	.010								
9	Risk aversion	5.73 (2.35)	.009	.050	‒.012	.043	.127	.108	‒.095	.005							
10	Experienced hire	48.10%	‒.315	.007	‒.012	‒.042	.019	‒.048	‒.067	‒.022	.027						
11	Job satisfaction	4.53 (1.77)	.046	‒.010	‒.005	.076	.196	‒.034	.083	‒.058	.065	‒.027					
12	Relationship with supervisor	3.53 (1.07)	‒.008	.040	.050	.036	.169	.032	‒.031	‒.075	.098	.038	.329				
13	Average weekly miles	1,715.30 (675.43)	.163	.231	‒.259	.331	‒.058	.013	‒.002	‒.063	‒.027	.070	‒.068	‒.029			
14	Performance (MPG benchmark)	80.06%	.081	‒.014	.046	.400	‒.008	‒.062	.117	‒.003	‒.043	‒.034	.074	‒.045	.077		
15	Performance (Accident)	13.1%	‒.134	‒.045	‒.003	‒.074	.010	‒.018	.001	.033	‒.016	.109	.090	.002	.029	‒.029	
16	Turnover	26.4%	‒.252	.118	.054	‒.146	.026	‒.026	‒.005	.076	‒.026	.052	‒.112	‒.075	‒.057	.063	.093

Note: All correlations greater than.070 are statistically significant at *p* < .05

### Measures

***Voluntary* *turnover.*** The dependent variable in our analyses was whether a driver voluntarily left the organization in the six months following the survey. Of the 711 drivers who completed the survey, HR records indicated that 188 (26.4%) left the company within six months after the survey. In this regard, Trucking Co. has comparable turnover to the industry average (25.5%; [79]). Trucking Co. offers both full truck load services (e.g., dedicated service lines) as well as less-than-truckload (LTL) services (i.e., multiple shipments in one truck). The company has a lower turnover rate than the average for full truck load companies (41.5% for 6 months) but a higher turnover rate than the average for LTL companies (9.5% for 6 months). However, a comparison of the turnover rate in our sample to the average of both types of organizations reveals that turnover in our sample is representative of the turnover that would be expected from a mix of full truck load and LTL drivers (26.4% for 6 months vs. 25.5% for 6 months). Those who left Trucking Co. in the six months following survey data collection were employed for an average of 60.9 (SD = 132.7) weeks.

***Pay*** ***average and* *v******ariability.*** For each driver, we calculated the within-person average and standard deviation of weekly paycheck amounts for the period of observation using the HR records. Whenever a driver did not work for a week due to vacation or sick leave, the paycheck was removed from the calculation of the average and standard deviation instead of being included as a 0. We opted to use the standard deviation as the measure of pay variability following prior work in the literature [27,31]. Alternative models using the coefficient of variation provide similar findings except for self-efficacy, which is in the hypothesized direction but not statistically significant as a moderator. Among those who started working with the company during the observation period (N = 211, 21.8%), pay average and variability was calculated between the hire date and the end of observation or turnover date. Analyses without these drivers led to similar results as those reported in the paper using the full sample. On average, drivers earned $1,049.34 (SD = $268.71) per week, but with substantial differences in the standard pay deviation (M = $282.20, SD = $111.19). The average estimated annual pay among drivers was $52,012, which is above the national median wage for commercial truck drivers of $48,310.

**Fig 2:**
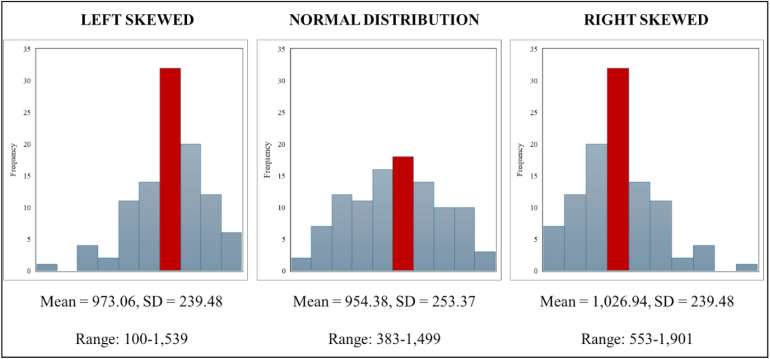
Illustration of Pay Skewness.

***Shape of*** ***paycheck* *d******istribution.*** We measured the direction of pay variability for each worker by calculating the skewness of their pay distribution. Skewness captures the relative frequency that a driver’s paychecks fall above or below their average paycheck. It was calculated using the sample size-adjusted Pearson-Fisher coefficient of skewness [80] of each drivers’ paycheck distribution. This measure of skewness equals 0 for a normal distribution, contains a negative value when the paycheck distribution is skewed left (i.e., the majority of drivers’ paychecks fall above the average of their distribution) and contains a positive value when the paycheck distribution is skewed right (i.e., the majority of drivers’ paycheck fall below the average of their distribution). Whereas a right-skewed paycheck distribution indicates that paychecks more frequently fall below the average paycheck, a left-skewed paycheck distribution indicates that paychecks more frequently exceed the average paycheck. Fig 2 provides a visual representation of how the skewness will capture individual differences in the shape of the paycheck distribution among drivers with a comparable pay average and variability. On average, the paycheck distribution from drivers in our sample was slightly left-skewed (M = −.048, SD = 1.05). This can be interpreted as that 38.6% of the average driver’s paychecks fell below their mean paycheck amount.

***Performance*** ***i******ncentives.*** The extent to which variable pay was driven by performance-based incentives vs. non-performance factors was measured by the average proportion of drivers’ paychecks that was derived from performance incentives. Drivers within our sample worked under numerous incentive plans depending upon their specific business unit and when they joined Trucking Co. Common criteria within these incentive plans included fuel efficiency, days worked, overspeed, accidents and violations, and number of shipments transported per day. Performance pay was calculated based on meeting the weekly benchmarks for the criteria included in a driver’s incentive plan, which were adjusted for the specific loads and trips that drivers were assigned within the week. While we did not have access to the details of each driver’s incentive plan, we were able to calculate the average proportion of a paycheck that originated from performance incentives versus non-performance factors for each driver. On average, 3.26% (SD = 2.29%) of drivers’ weekly paychecks originated from performance-based incentive payments.

***Self-efficacy*.** Self-efficacy was measured on the survey using the scale developed by Chen, Gully, and Eden [81]. The scale captures agreement with eight statements including “I will be able to achieve most of the goals that I have set for myself” and “When facing difficult tasks, I am certain that I will accomplish them” on a scale from 1 (*Strongly Disagree*) to 7 (*Strongly Agree*). Responses were internally consistent within our sample (Cronbach’s α = .930).

***Paycheck*** ***d******ependence.*** Each survey respondent was asked to indicate the proportion of their total household income that comes from their work with Trucking Co. in ten-percent intervals from 10 percent to 100 percent. Drivers reported that, on average, their paycheck comprised 79.7% (SD = 22.84%) of their total household income with a range from 30 to 100 percent. In this measure, 100% means that there are no other sources of household income. Approximately half (42.99%) of the drivers in the sample derived their full household income from their paychecks and 75% derived at least 70% of their household income from their paycheck, indicating that most were the breadwinner in their household. No drivers in the sample indicated that their paycheck was less than 30% of their household income. Alternative analyses using a binary measure representing (1 = only source of income, 0 = other sources of income) led to similar results as reported.

***Control*** ***v******ariables.*** All analyses included a series of control variables purposefully selected to account for alternate explanations. First, we controlled for the presence of a household partner or spouse. In our sample, roughly two-thirds of drivers had a household partner. Second, we accounted for whether a driver had prior commercial trucking experience. At Trucking Co., there are two pathways to entry: Drivers were classified either as a novice hire who earned their commercial driver’s license (CDL) through Trucking Co. or as an experienced hire who was already licensed. Those who acquired their CDL through Trucking Co. may have a higher level of pay variability because they have less experience with effective strategies to mitigate fluctuations in earnings but also may be more reluctant to leave because of Trucking Co’s investment in their certification. In our sample, 48% of drivers were classified as experienced hires. Third, we included drivers’ satisfaction with their job and their direct supervisor since prior research suggests that the relationship between pay variability and voluntary turnover may be influenced by being overall unsatisfied with one’s job or having a poor relationship with one’s supervisor [82]. Job satisfaction (M = 4.53, SD = 1.77) was measured by a one-item measure asking drivers to indicate their level of satisfaction on a scale from 1 (*Extremely Dissatisfied*) to 7 (*Extremely Satisfied*). The relationship with their supervisor (the business unit leader in their home location) was measured by the eight-item leader-member exchange scale published by Graen and Uhl-Bien [83] which included items such as “How well does your manager understand your job’s problems and needs?” and “What are the chances that your manager would use his or her power to help you solve a problem in your work?” on a 5-point scale (M = 3.53, SD = 1.07). These items had good internal consistency (Cronbach’s α = .930). Fourth, we accounted for individual differences in risk aversion given that these differences are likely to determine voluntary turnover [82,83] and how much one can tolerate uncertainty in earnings [84–86]. Risk aversion was measured by asking respondents to indicate on a scale from 1 (*Completely Unwilling*) to 10 (*Completely Willing*) how willing they were to take risks in their life‌‌ (M = 5.73, SD = 2.35).

Finally, we controlled for drivers’ workload and performance using data from the HR records. Driver performance was included to account for the possibility that the relationship between pay variability and voluntary turnover was attributable to low performers. Some scholars have suggested that high performers may be less likely to leave in response to variable compensation because they can earn more (e.g., [87]). Thus, we wanted to examine if the relationship between pay variability and voluntary turnover existed beyond performance differences. Performance in this context was captured by drivers’ attainment of their MPG benchmark, a common industry metric [88]. We supplemented the attainment of the MPG benchmark with an indicator of whether drivers incurred preventable accidents within the period of observation given that road safety is another key performance metric [56]. On average, drivers in our sample attained their MPG benchmark 80% (SD = 16.42) of the weeks under observation and 13% experienced a preventable accident. We also controlled for drivers’ workload, measured by the average miles driven per week to account for the possibility that those who drive for longer periods of time may have greater pay variability and/or a higher likelihood of voluntary turnover. Driving longer distances creates a higher likelihood for outside factors such as weather and traffic to influence pay variability and these drivers may be more likely to leave because they are away from home for longer periods of time [89]. Drivers drove an average of 1,715.30 miles (SD = 675.43) per week.

### Analytic approach

To test our hypotheses, we used a series of Cox proportional hazards regression models ([90]; see [91] for application to turnover) to examine whether individual differences in pay variability are associated with the risk of voluntary turnover. Afterwards, we examined the moderating effects of pay skewness, perceived control through performance incentives and self-efficacy, and paycheck dependence on this relationship. All models reported here included a fixed effect for a driver’s line of business (e.g., dedicated service lines, less-than-truckload) to account for differences in the nature of the work such as time away from home as well as robust standard errors to account for heteroscedasticity [92].

We use a between-person comparison rather than within-person changes in pay variability over time [31] for conceptual and empirical reasons. From a conceptual perspective, our qualitative data suggests a significant challenge with capturing a within-person relationship between pay variability and voluntary turnover. That is, as observed in our exploratory qualitative data, people may not respond immediately to pay variability by leaving [93] because the disruption of pay variability to their lives may build up distress over time and/or workers may try to use alternative strategies to mitigate the variability but only eventually leave when they feel they have exhausted their options. By using a between-person analysis, we analyze the relationship between pay variability and turnover without requiring assumptions about the lag between an increase in the variability of a driver’s pay and leaving the company. Empirically, we opted for a between-person comparison because incorporation of the survey data to capture the moderator variables limits observation of within-person changes in pay variability over time. Incorporation of the survey data collection meant that we only were able to retrieve data from those who were employed at the time of the survey and consented to participate. We also only had access to turnover records for six months after the survey. As such, a between-person analysis facilitates the use of the survey measures in testing the contextual moderators.

## Results

**Fig 3:**
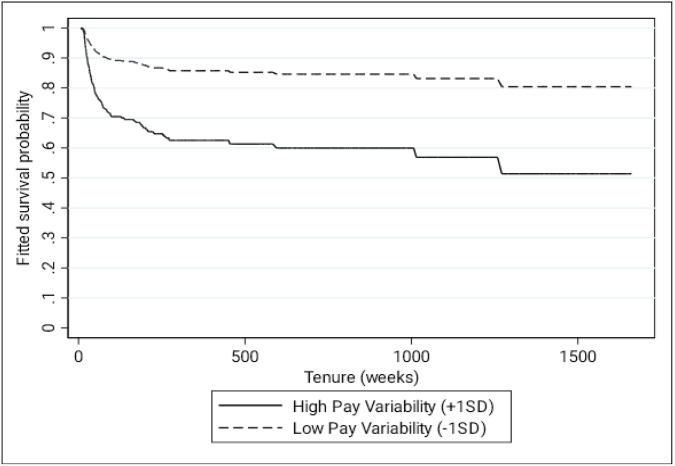
Fitted Survival Probability by Individual Differences in Pay Variability.

**Fig 4:**
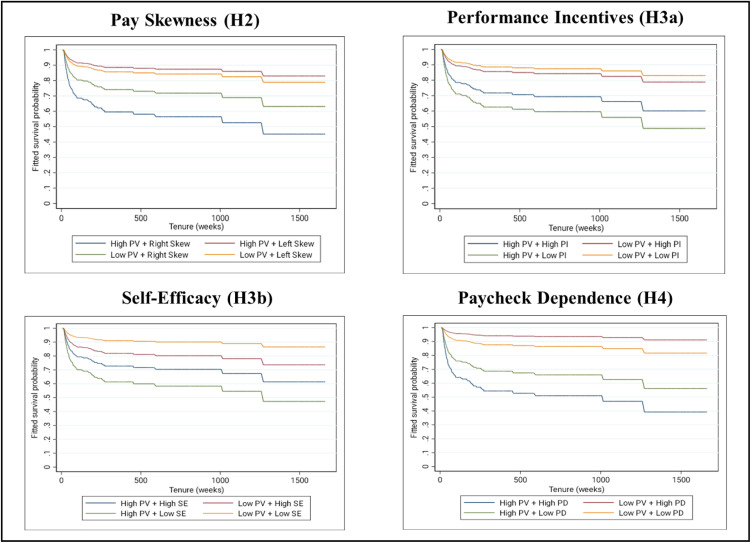
Fitted Survival Probability by Individual Differences in Pay Variability (PV) and the Contextual Moderators.

Table 2 depicts the results of our main analyses. In support of Hypothesis 1, we found that pay variability was positively associated with the likelihood of voluntary turnover (β = .5582, *p* < .001; see Fig 3). All else equal, a one standard deviation difference in pay variability was associated with a 13.6% increase in the probability of voluntary turnover. Thereafter, we examined the moderating influence of skewness, performance incentives, self-efficacy, and paycheck dependence on this relationship (see Fig 4). We found that the skewness of a driver’s paycheck distribution significantly influenced the relationship between pay variability and turnover (β = .1879, *p* = .021). Pay variability had a stronger positive relationship with voluntary turnover from Trucking Co. among drivers with a right-skewed paycheck distribution where paychecks were more frequently falling below their average (+1SD: β = 1.050, *p* < .001) compared to drivers with a left-skewed paycheck distribution where paychecks were more frequently exceeding their average (−1SD: β = .6557, *p* < .001).

**Table 2:** Cox Regression Models Estimating Risk of Voluntary Turnover.

Variables	Model 1	Model 2
*Pay variability (H1)*	.5582*** (.0551)	.5273*** (.1073)
*Shape of paycheck distribution (H2)*		
Pay skewness	−.0447 (.0551)	.0730 (.1109)
Pay variability x Pay skewness		.1879* (.0814)
*Perceived control (H3)*		
Performance incentives	−.2196+ (.1323)	−.0431 (.1398)
Self-efficacy	.1037 (.0851)	.0832 (.0841)
Pay variability x Performance incentives		−.2593* (.1170)
Pay variability x Self-efficacy		−.3067** (.1079)
*Paycheck dependence (H4)*		
Paycheck dependence	.5582*** (.0018)	−.0033 (.0054)
Pay variability x Paycheck dependence		.0138** (.0046)
Paycheck average	−.0040*** (.0004)	−.0045*** (.0004)
Household partner	.3019 (.1841)	.2494 (.1932)
Multiple jobs	.6477+ (.3800)	.8910* (.3879)
Risk aversion	.0174 (.0323)	−.0013 (.0325)
Experienced hire	.0142 (.1596)	.2055 (.1647)
Job satisfaction	−.1009* (.0432)	−.0763+ (.0444)
Relationship with supervisor	−.1296+ (.0724)	−.1107 (.0731)
Average weekly miles (ln)	−.3382* (.1457)	−.3519* (.1514)
Performance (MPG benchmark)	1.884*** (.5007)	2.258*** (.5288)
Performance (Accident)	.6725** (.1958)	.5995** (.1995)
Line of business fixed effect	YES	YES
Observations	711	711
Failures	188	188
Log likelihood	−1046.09	−1024.76
LR chi-squared	219.05***	261.70***
Comparison to (1)		42.65***

***p < .001, **p < .01, *p < .05, + *p* < .1

Next, we examined whether higher levels of perceived control due to structure (performance incentives) or disposition (self-efficacy) attenuated the relationship between pay variability and voluntary turnover. In support of Hypothesis 3a, we found that the relationship between pay variability and turnover depended upon the proportion of a driver’s paycheck originating from performance incentives (β = −.2593, *p* = .027). Pay variability had a stronger positive association with the likelihood of voluntary turnover when drivers derived a relatively smaller proportion of their paycheck from performance incentives (−1SD: β = .9963, *p* < .001) compared to when performance incentives constituted a larger proportion of their paycheck (+1SD: β = .6955, *p* < .001). Consistent with Hypothesis 3b, we further found that the relationship between pay variability and voluntary turnover varied by differences in self-efficacy (β = −.3067, *p* = .004). Pay variability had a stronger positive association with the likelihood of voluntary turnover among drivers with lower levels of self-efficacy (−1SD: β = 1.139, *p* < .001) compared to drivers with higher levels of self-efficacy (+1SD: β = .5497, *p* = .001).

Finally, we investigated the moderating influence of paycheck dependence on the relationship between pay variability and turnover. Consistent with Hypothesis 4, we found a significant moderating influence of paycheck dependence on the relationship between pay variability and voluntary turnover (β = .0138, *p* = .003). Pay variability had a stronger positive relationship with the likelihood of voluntary turnover among drivers with high paycheck dependence (+1SD: β = .9963, *p* < .001) compared to those with low paycheck dependence (−1SD: β = .6955, *p* < .001).

### Supplemental quantitative analyses

We further leveraged our data to provide more insight into the implications of this relationship between pay variability and turnover by comparing stayers and leavers with pay variability below and above the median (see Table 3). First, we examined differences between stayers and leavers in their annual earnings relative to other truck drivers in their state to investigate the likelihood that drivers with high levels of pay variability are leaving predominantly for higher-paying jobs. We collected the median commercial truck driver annual pay for the state in which each driver resided from the Bureau of Labor Statistics and then calculated the difference in annual earnings between the drivers in our sample and the state median. On average, drivers in our sample earned 16.7% (SD = 31.7%) or $7,607.25 (SD = 14,769.37) more a year than the median driver in their state, indicating that Trucking Co. tends to pay higher wages on an annual basis than their local competitors. At high levels of pay variability, stayers (M = 1.29, SD = .29) earned 29% more than the median truck driver in their state but even those who left (M = 1.13, SD = .41) earned 13%, or approximately $6,000, more in a year than the average driver in their state. Notably, the most common sign-on bonus during our study was $1,500 [89], which is less than the monetary difference between the annual earnings of drivers with high pay variability and the median truck driver.

**Table 3:** Comparisons of Wage Premium, Performance, and Aptitude among Stayers and Leavers by Level of Pay Variability.

	*Low pay variability*			*High pay variability*		
	**Stayers**	**Leavers**	**Difference**	**Stayers**	**Leavers**	**Difference**
Relative premium over state median annual wage	1.14	.91	.230	1.29	1.13	.161***
Monetary premium over state median annual wage	6,778.60	−4,205.20	10,983.80***	13,562.64	5,865.37	7,697.27***
MPG goal	.799	.818	.028	.796	.823	.020
Accident	.118	.228	.110**	.103	.148	.045
Cruise control pct	.366	.353	.019	.392	.403	.007
Overspeed pct	.013	.015	.002	.013	.013	.000

***p < .001, **p < .01, *p < .05, + *p* < .1

Taken together, this comparison suggests that the relationship between pay variability and turnover is unlikely to be explained by drivers with high levels of pay variability moving to other jobs offering higher pay or a sign-on bonus. Most drivers with high pay variability in our sample who quit Trucking Co. were already earning more in a year than the median truck driver in their local labor market, consistent with the notion that workers have the potential for higher earnings under variable pay systems, and the most common sign-on bonus would not make up for the difference. As a result, drivers were likely sacrificing some of their aggregate earnings by leaving Trucking Co. for a competitor in the industry, trading off earnings for stability. Within the trucking industry, drivers may leave for an organization with hourly pay, an alternative pay schema used in the industry [56], or an organization that better calibrates load assignments to limit pay variability. Leaving for similar occupations (e.g., personal drivers) would also likely result in lower annual earnings because these occupations often pay less than the tractor-trailer truck driving being done by those in our sample (e.g., the average Uber driver earns $13.17 per hour, which corresponds to a roughly 44% reduction in annual wages for our sample of drivers; [94]). Indeed, the U.S. median wage for tractor-trailer truck drivers ($48,310) exceeds the median wage for jobs that require a high school education ($41,000), which was the most common highest level of education completed in our sample. Thus, while we cannot entirely rule out that drivers with a high level of pay variability are leaving Trucking Co. to increase their earnings or receive a sign-on bonus, our available data suggests that these are highly unlikely to explain the relationship between pay variability and voluntary turnover.

In addition, we explored the implications of turnover among drivers with higher levels of pay variability for Trucking Co. While research suggests that turnover is often costly for organizations [95], some scholars have argued that turnover can be functional when it is poor performers who are disproportionately more likely to leave [82]. This means that lower-performing drivers may be more likely to experience high levels of pay variability, and thus sorting out these drivers for Trucking Co. We further examined this in Table 3 by comparing stayers and leavers on two performance measures (MPG attainment and preventable accidents) and two measures of driving skill used within Trucking Co. and the broader trucking industry (amount of driving time surpassing the speed limit and using cruise control). There was no significant difference in the performance measures between stayers (MPG goal: M = .80, SD = .16; accident: 10.3%) and leavers (MPG goal: M = .82, SD = .15; accident: 14.8%) among drivers with a high level of pay variability. Additionally, we found no significant difference in driving skill between stayers (overspeed: M = .01, SD = .03; cruise control: M = .40, SD = .17) and leavers (overspeed: M = .01, SD = .03; cruise control: M = .40, SD = .15) with high levels of pay variability. Hence, we found no evidence that only lower-performing or less skilled drivers were sorting out due to higher levels of pay variability, which indicates that the relationship between pay variability and voluntary turnover observed within our sample is unlikely to be functional for Trucking Co.

### Supplemental qualitative analyses

We conducted supplemental analyses on qualitative data from our truck drivers to provide additional evidence for the mechanisms underlying the relationships observed in our results. At the end of the survey, drivers responded to three open-ended questions about their pay (“What is your opinion about the amount and structure of the pay you receive at Trucking Co.?”), what was most important in the work (“What does it take to be a successful driver at Trucking Co.?”), and future plans (“If you are considering leaving Trucking Co. in the near future, what is the main reason behind this decision?”). Given our interest in pay variability, the first author and a research assistant, blind to the results of the quantitative data, coded responses (N = 1,155) for those with a level of pay variability above the sample’s median. We coded these responses in categories that matched our quantitative measures. Responses averaged seventy words, or roughly 5.6 sentences.

The open-ended responses supported the findings from the quantitative analyses. Among the drivers considering leaving Trucking Co., the majority (57%) reported that pay variability was the main factor. Drivers with high levels of pay variability noted the uncertainty in their earnings, characterizing their pay as “very unstable” (76), “having huge fluctuations” (691), and “really inconsistent” (253) because “no one knows what they will make from week to week” (991). This uncertainty was an adverse experience for drivers as some reported “nerve-wracking” (233) distress and having limited influence over their weekly earnings – “The first week I made $1,400–- [the] second and third [week I] made around $300-400” (273). Due to factors outside of their control, such as weather, paperwork errors and breakdowns, drivers felt they were unable to control the amount they earned with each paycheck, leading them to appraise the variability as an aversive experience. “Financial hell” is what one driver called his experience at Trucking Co., and when describing the explicit reasons he was contemplating leaving, he noted: “Sometimes loads get canceled and it is a struggle trying to make up the miles from a canceled load and if this continues, I might have to find new employment. I am in financial hell right now and trying to stay positive and I love my job, but I cannot pay my bills when loads get canceled” (440). Other drivers attributed the fluctuations in their earnings to fluctuations in market demand. As one driver said, “business has been slow and my paychecks are becoming very unpredictable… my colleagues at other companies are not experiencing this, which makes me believe that we are losing business to competitors” (755). Noting the relationship between loads offered and pay, another driver who was considering leaving said, “We do not have consistent miles hence we don’t have consistent pay” (793). In sum, a constant theme throughout the qualitative data was that pay variability represented an aversive experience for this population of low- and middle-wage workers.

Regardless of the outside factors drivers ascribed to their pay variability, their perceived lack of control over their paychecks led them to contemplate leaving Trucking Co. because it threatened their ability to provide for their households. A driver considering quitting said, ‘I shouldn’t have to worry constantly if I’m going to be able to make a mortgage payment month after month or put food on my table. I struggle week after week, month after month, to be a man and support my family” (972). Yearning for dignity, another said, “If I was to leave Trucking Co. after 21 years here… [it’s so] I can budget properly and live like a decent human being” (891). Similarly, a twenty-year plus veteran who was planning to quit stated, “I can’t plan for anything financially at Trucking Co., because I have no idea what money I’ll make next week or the week after” (853). Noting his immediate resignation, a driver said he was “disappointed with the fucked-up pay… FUCK YOU TOO Trucking Co., I’m out of here ASAP” (150). Consistent with the quantitative findings, the qualitative data emphasizes that high pay variability was appraised as a threat to drivers and, hence, pushed many towards leaving. despite being satisfied with the work.

The supplemental qualitative data provided support for the moderating conditions surrounding perceptions of control. Differences in performance incentives and self-efficacy emerged from the qualitative data as central to how drivers experienced and coped with pay variability. Foremost, several drivers with a lower percentage of their paycheck derived from performance incentives explicitly mentioned an intention to quit. One driver noted that his paychecks did not reflect the effort he put into his work and said, “I am going to go [leave] because I feel like I deserve more cents per mile and better paying loads because I never call off work, I work all my days. I am a great, safe driver” (746). In contrast, workers with a relatively higher-than-average percentage of their paychecks coming from performance incentives often reported they were “happy with the pay” (279) because they were paid “fairly for the amount and type of work that [they do]” (805) and that Trucking Co.’s incentives were “far above most” other companies (633). These drivers noted their intention to stay, stating that they “love working at Trucking Co.” (925) and planned to stay until retirement, in part, because they “couldn’t ask for more” (957). Differences in self-efficacy were also crucial in whether drivers believed they could successfully manipulate their paychecks. When asked to describe what made for a successful driver, they noted one needed to be “self-motivated and plan for different things” (476) and able to “accomplish tasks independently” (857) and “adapt to different situations and problems, because something will not go right pretty much every day” (930), all of which indicate higher levels of self-efficacy. One driver credited his success to, “being resourceful and having ingenuity to solve your own problems - i.e., fixing a broken crank handle, so you don’t have to call and wait on third party maintenance [losing paid driving time]” (133). In sum, the qualitative data supported the importance of workers’ perceived ability to affect their paychecks through their own behaviors which, in turn, influenced the relationship between pay variability and voluntarily turnover.

Last, the qualitative data provided additional evidence that the economic composition of drivers’ households shaped the experience of pay variability. A driver whose income supported his family of three was frustrated because he could not “rely on one constant [paycheck] number each week which ultimately result[ed] in shortages in my family’s budget’ (258). This is consistent with studies showing that pay variability can trigger spells of episodic poverty where the household does not have enough resources to meet its needs [42], particularly among low- and middle-wage workers [20]. The stay-at-home wife of another driver was forced to “get a job to help make ends meet” (315) to support their two children due to fluctuation in his earnings. Those who were unable to rely on other household members’ steady income frequently mentioned thinking about quitting. One driver said he had just put in his termination paperwork to find steadier work that allowed him to better care of his family (740) while others were considering leaving “for more stability for my family” (853) because of the difficulty in meeting regular expenses such as their mortgage (973). Other drivers depended on second jobs to support their households; “I love my job as a company driver, but if this was my only source of income I would have been gone” (519). Alternatively, some drivers noted that they were only able to cope with pay variability and remain at Trucking Co. because others were not dependent upon their paycheck. One driver said that the only reason he could stay at Trucking Co. was because he “was single without family obligations” (700). Likewise, another driver said, “My pay has huge fluctuations every week [and I have the] privilege of having no debt, children, spouse, or car/house payments. If I had any of those things, I would be more frustrated with the pay structure. It is still frustrating, nonetheless.” (888). Overall, the qualitative data supported the quantitative findings in that paycheck dependence enhanced the extent to which pay variability was appraised as a threat that workers needed to distance themselves from.

## Discussion

As employers shifted risk away from themselves onto individual workers [4,6], it had profound implications for employment relationships and workers, notably in the rise of variable compensation systems [1,34,96,97]. Emerging research suggests that pay variability originating from these systems can be detrimental to low- and middle-wage workers’ lives (e.g., [22,27]) and increase their propensity to leave their jobs [31]. Using multi-sourced qualitative and quantitative data from two jobs, we extend this literature by examining divergence in the experience and response to pay variability using identifying new moderators.

We first explored low- and middle-wage workers’ experiences of pay variability using semi-structured interviews and a survey with gig workers (ride-hailing drivers), a group prone to experiencing pay variability. These workers experimented with strategies to mitigate the variance in their paychecks, such as chasing incentives. However, when they felt unable to effectively control their earnings through these strategies and/or alternative household income were not available as a safety net for shortfalls, they were forced to work longer hours, increasing strain and dislike for their work, or distanced themselves by taking other jobs. Drawing from these insights, we developed a conceptual model rooted in cognitive appraisal theory (CAT; e.g., [32]) to outline critical factors shaping workers’ experiences of pay variability, and thereby leading to individual-level heterogeneity in voluntary turnover. Consequently, we predicted that higher levels of pay variability are more likely to result in voluntary turnover among workers whose paychecks are more frequently falling below their average, who do not believe they can control their earnings, and whose households are highly dependent upon their paycheck.

Using matched survey and archival data from full-time truck drivers, we found a positive relationship between pay variability and voluntary turnover. However, consistent with our theoretical framework based on CAT, pay variability had a stronger positive association with voluntary turnover among drivers whose paychecks more often fell below their average paycheck, whose earnings were less influenced by performance incentives, who had low levels of self-efficacy, and whose households were highly reliant on their paycheck. Supplemental quantitative analyses indicated that the observed relationship between pay variability and voluntary turnover is unlikely to be explained by a competitor’s higher wages or a sign-on bonus and unlikely to be functional as drivers with high levels of pay variability who decided to leave were not disproportionately low performers or less skilled. Supplemental qualitative analyses offered additional support for our arguments and identified threat appraisals as the underlying mechanism.

It is possible that our findings with the truck driver data may reflect pay variability becoming intensified due to lower effort or attention as workers anticipate their organizational exit. We explore this with our data by examining changes in truck driver behavior in the six weeks prior to observing turnover, finding no evidence consistent with decreased effort or attention in the weeks prior to leaving. There was no difference in the performance pay earned by drivers in these six weeks (*B* = −.3066, *p* = .956) and workers perform better on average with a higher likelihood of hitting miles per gallon goals (*B* = .1463, *p* < .001) and lower likelihood of an accident (*B* = −.0046, *p* < .001). There was no difference in speeding (*B* = .0084, *p* = .242), further demonstrating that drivers were being attentive and remained motivated to earn performance incentives which were tied to their speeding. While there is lower non-performance pay for loads in this period (*B* = −206.79, *p* = .013), but much of this appears to be driven by workers using accumulated time off being used prior to leaving. While we are cautious in interpreting these results, these findings suggest that drivers are reducing their effort or attention in the weeks before leaving, suggesting that reverse causality is unlikely.

### Theoretical contributions

Eschewing the “extremely narrow” employer-centric perspective that dominates the literature on work practices [18], we contribute to an emerging stream of research highlighting the potential costs of variable compensation systems for workers (e.g., [17,98]). Variable compensation is commonly viewed as positive for workers due to the opportunity to earn more when they attain performance criteria and/or when there is high demand for goods and services. At the same time, economic models suggest that workers will effectively insulate themselves from downward fluctuations by developing savings, adjusting consumption, or diversifying their streams of income [52]. Yet, a growing number of studies suggest that pay variability originating from these systems is an aversive experience for workers (e.g., [22]), particularly for those at the low and middle end of the income distribution living paycheck-to-paycheck. Recent research by Conroy et al. [31] suggests organizational costs associated with workers’ experience of pay variability by increasing the likelihood of voluntary turnover.

In this paper, we built upon this research to provide an in-depth examination of pay variability among low- and middle-wage workers and uncover factors that may moderate their experiences and response. Leveraging our qualitative data across two samples, we explain this relationship by documenting a strong aversion to pay variability due to the threat of earnings being insufficient to meet their household’s financial needs which, in turn, motivate turnover. Further, we identified important contextual determinants—the shape of the paycheck distribution, feelings of control, and paycheck dependence—that produced differences in the extent to which workers appraised pay variability as a threat, introducing nuance into the relationship between pay variability and turnover. By identifying these contextual moderators, our paper suggests that organizations can allay financial precarity under variable compensation systems, and thus have some ability to mitigate the relationship between pay variability and voluntary turnover.

Our findings further suggest that the use of variable compensation for low- and middle-wage jobs is not just harmful for workers but can also be costly to organizations. When a high level of pay variability is appraised as a threat, it motivates people—even the highest performing and most skilled workers—to leave. When compared to stayers, leavers with high levels of pay variability in our sample did not significantly differ in their performance or driving skill, indicating that the company lost valuable human capital. In addition, this turnover can also be costly because churn carries replacement costs and introduces operational disruptions [95]. As an illustration of these costs, we estimated the turnover cost of pay variability for Trucking Co. based on the effect size in our sample and the average replacement cost for a commercial truck driver ($8,234; [99]). We calculate that a 1SD increase in pay variability could be associated with approximately $9.7 million per year in turnover costs due to an additional 1,178 drivers leaving Trucking Co. per year. Thereby, our research shows that there are long-term costs for employers associated with transferring market risk onto individual workers through variable compensation systems.

### Practical implications

This paper offers insight into the prerequisites for variable compensation to be less harmful, and possibly more desirable, for low- and middle-wage workers. Indeed, there is urgency to understand the consequences of these systems given the trend towards more frequent paychecks (i.e., daily pay) among these workers [29,30] and the significant number of people struggling to manage pay variability within their full-time jobs [20,28]. First and foremost, our findings indicate that employers need to design their compensation systems to limit market-based volatility (e.g., customer demand). Variable inputs into earnings must be isolated from factors that are outside of workers’ control and there should be clarity in the requirements for achieving higher earnings. While some jobs do offer regular overtime to smooth pay variability, as we saw in the case of the ride-hailing drivers, spending more time at work can increase mental and physical strain fueling disgruntlement and fatigue. Alongside these changes, organizations can also implement interventions to positively impact workers’ self-efficacy or beliefs about their ability to meet the performance standards of the job [100,101]. In both our samples, we found that workers felt less threatened by pay variability when they were confident in their ability to control some of the volatility in their earnings. For truck drivers, for example, companies might offer basic mechanic courses to give drivers the ability to take care of any minor issues that may arise that would minimize down-time and provide them with a sense of control.

Second, employers can limit the threat of pay variability by instituting pay bands or minimum pay amounts to insulate from downward fluctuations. By receiving a paycheck with a base amount, workers can plan around their financial obligations, decreasing the potential for missing bill payments or food insecurity. While variable compensation systems are the predominant pay schema in the trucking industry, a small and growing segment of the industry is offering hourly pay based on tenure [56]. In addition, employers incentivize building a safety net through employee savings programs for when paychecks are lower than usual [102]. Organizations could also subsidize or sponsor membership to apps such as Evenly, Trezeo, and Portify, which create savings accounts that are automatically added to or subtracted from based on each paycheck to smoothen volatility from paycheck to paycheck.

Finally, organizations that deploy variable compensation systems should integrate tools that allow workers to foresee and manage their pay, such that fluctuations are not a surprise. Workers complained about the lack of pay transparency: truck drivers noted Trucking Co.’s hundreds of pay plans and ride-hailing drivers were unsure about how the algorithmic manager determined the rates. Increasingly, algorithms undergird variable compensation systems, deciding when and how much workers are compensated; the secrecy surrounding the algorithms make it nearly impossible for workers to know what their paychecks will be ([103-105]). This algorithmic opacity coupled with pay variability can increase turnover, especially for gig workers [106]. Initiatives which promote pay transparency, such as Instacart’s batch pricing system, in which workers are shown base pay, tips, and any incentives before accepting a job, can smooth workers’ pay and attenuate turnover [107,108].

More broadly, this research contributes to broader debates on workplace insecurity and structural precarity by identifying pay variability as a central institutional mechanism through which economic risk is distributed from employers to workers. While precarity is often associated with nonstandard work arrangement or persistently low wages, our results demonstrate that pay volatility itself constitutes a distinct and consequential dimension of workplace insecurity and structural precarity. Elevated turnover among workers experiencing high pay variability suggests that volatility, rather than earnings alone, undermine job retention. This pattern aligns with research that accounts for precarity as a systematic feature of contemporary labor markets (e.g., [39,97,109]) in which employers maintain flexibility by externalizing uncertainty and individualizing risk even within standard work arrangements. The implications of these findings extend to other industries and occupations, as pay variability is increasingly prevalent in some of the fastest-growing jobs such as health care staffing, logistics, commission-based work and scheduling-driven service jobs, By linking pay variability to turnover, this study extends research on how compensation structures themselves contribute to the reproduction of insecure employment and reinforce patterns of labor churn. Those in jobs exposed to pay variability are faced with the decision of enduring uncertainty in their income or find a lower-paying job that provides them with some stability. Future research should consider how this economic risk interacts with other forms of risks (e.g., physical, environmental) that are being placed on workers.

### Future research directions

Our research offers several avenues for future research on variable compensation and pay variability. First, research on pay variability has often focused on occupations that consists mostly of White men who are their household’s primary breadwinners and therefore may feel increased psychological pressures to provide a stable household income [110]. Given the importance of the economic composition of workers’ household to the experience of pay variability identified, future research should explore how different demographic groups that face less pressure to provide for their families (e.g., White women) respond to pay variability. Second, our paper focus on the lived experiences of low- and middle-wage workers, which raises the question of whether our findings apply to higher-wage workers as well. Pay variability could be disruptive to meeting one’s financial needs even at higher income levels, but they are more likely to have savings and/or multiple streams of income to absorb financial shocks and could limit discretionary spending to absorb downward fluctuations [69]. This suggests that there may be an income threshold where pay variability becomes less disruptive for workers. Future research could explore the experience of pay variability among higher-wage worker populations whose paychecks are mostly derived from variable sources (e.g., luxury real estate agents, high-yield stock traders) and explore whether there is a threshold at which pay variability becomes an adverse experience. Third, there may be other characteristics of fluctuations in earnings that may influence the experience of pay variability, such as the timing between paychecks and relative size of a single fluctuation. Future research could also investigate variation in the characteristics of fluctuations between paychecks to further unpack the conditions under which pay variability is most likely to represent an adverse experience for workers and motivate turnover.

Lastly, there are several limitations to this study that provide opportunities for future research. While we have included several control variables and can show that reverse causality is unlikely, our study is unable to establish a causal relationship between pay variability and turnover. Alternative study designs – such as field, natural, or lab experiments – can provide more evidence for causality. Moreover, our data did not provide detailed information about drivers’ incentives plans and other work characteristics. More comprehensive data sets could provide additional insights about these potential moderators to the relationships documented ‌‌here.

## Conclusion

While variable compensation systems are often described positively for both organizations and workers, we observed in this mixed-method study that these systems can be highly disruptive for workers in low- and middle-wage jobs because pay variability can threaten their earnings falling short of their household’s financial needs. As a result, many become motivated to distance themselves from the job through voluntary turnover. Our paper has offered a theoretical framework to predict individual differences in workers’ aversion to pay variability, thus providing insight into how variable compensation systems may be redesigned to be less harmful for this population. Hence, we offer a roadmap towards variable compensation systems that can be beneficial for both employers and workers.

Thank you to the drivers at RideHail and Trucking Co. in sharing their stories. We extend special thanks for the constructive comments from the editor and the two reviewers. Previous versions of this manuscript benefited from insightful feedback from Matthew Bidwell, Peter Cappelli, Seth Caranahan, and Jon Jachimowicz. Deep gratitude to Michelle Borges, Sean Dew, Eli Gonzalez, Vidisha Hermani, Kalie Mayberry, Brandon Nguyen and Ruiling Wen for their research assistance. We are grateful for the editorial suggestions of Kristin McGuire and Timil Jones.
